# Metformin combined with local irradiation provokes abscopal effects in a murine rectal cancer model

**DOI:** 10.1038/s41598-022-11236-2

**Published:** 2022-05-04

**Authors:** Mineyuki Tojo, Hideyo Miyato, Koji Koinuma, Hisanaga Horie, Hidenori Tsukui, Yuki Kimura, Yuki Kaneko, Hideyuki Ohzawa, Hironori Yamaguchi, Kotaro Yoshimura, Alan Kawarai Lefor, Naohiro Sata, Joji Kitayama

**Affiliations:** 1 Department of Gastrointestinal Surgery, Jichi Medical University, Yakushiji 3311-1, Shimotsuke, Tochigi 329-0498 Japan; 2 Department of Clinical Oncology, Jichi Medical University, Shimotsuke, Tochigi Japan; 3 Department of Plastic Surgery, Jichi Medical University, Shimotsuke, Tochigi Japan

**Keywords:** Cancer, Immunology, Gastroenterology, Oncology

## Abstract

Although preoperative chemoradiation therapy can down-stage locally advanced rectal cancer (LARC), it has little effect on distant metastases. Metformin exerts an anti-cancer effect partly through the activation of host immunity. LuM1, a highly lung metastatic subclone of colon 26, was injected subcutaneously (sc) in BALB/c mice and treated with metformin and/or local radiation (RT). Lung metastases and the primary tumors were evaluated and the phenotypes of immune cells in the spleen and lung metastases were examined with flow cytometry and immunohistochemistry. Local RT, but not metformin, partially delayed the growth of sc tumor which was augmented with metformin. Lung metastases were unchanged in metformin or RT alone, but significantly reduced in the combined therapy. The ratios of splenic T cells tended to be low in the RT group, which were increased by the addition of metformin. IFN-γ production of the splenic CD4(+) and CD8(+) T cells was enhanced and CD49b (+) CD335(+) activated NK cells was increased after combined treatment group. Density of NK cells infiltrating in lung metastases was increased after combination treatment. Metformin effectively enhances local and abscopal effects of RT though the activation of cell-mediated immunity and might be clinically useful for LARC.

## Introduction

Neoadjuvant radiotherapy (RT) and systemic chemotherapy can induce downstaging of locally advanced rectal cancer (LARC) leading to reduced local recurrence rates^1–3^ and is now considered standard treatment for patients with LARC worldwide^4,5^. However, the incidence of distant metastases is not decreased, and many radiosensitizers have been examined in clinical trials to improve the efficacy and tolerability of RT.

Metformin is an oral antihyperglycemic drug used as first-line treatment for some patients with type 2 diabetes mellitus for over 50 years. Numerous epidemiologic studies have suggested that metformin use can significantly reduce the incidence of cancer development and mortality among patients with type 2 diabetes mellitus, although the rate of reduction differs for various types of cancer^6–11^. Numerous studies have demonstrated that metformin enhances DNA damage of tumor cells via multiple mechanisms which can exert radiosensitizing effects^12–17^. Since metformin has been widely prescribed because of its well-known safety profile and low cost, many clinical studies have already been conducted. Some of these studies have shown that metformin improves outcomes in patients who received RT for the treatment of various cancers including colorectal cancer^18–22^. However, synergistic effects of these two treatment modalities were not always evident^23–25^ and detailed molecular mechanisms of the synergism are still unclear.

Generally, it is believed that RT induces transient immunosuppression. However, multiple reports have suggested that tumor cells which are dead or dying after treatment with RT can present tumor-associated antigens to host immune cells and evoke innate and adaptive immune responses^26,27^. Therefore, many preclinical studies have been conducted on the synergistic effects between RT and immunotherapy using immune checkpoint inhibitors, which suggest that the anti-tumor effects of RT are further enhanced by the concurrent administration of antibodies to CTLA-4 and PD-1^28–30^. Some clinical trials also have suggested synergistic effects between RT and recently approved antibodies against PD-1 and CTLA-4^31,32^. However, such benefits have not been confirmed in other clinical studies^33,34^, and the nature of agents which optimize responses to RT remain to be elucidated.

Recent studies have demonstrated that metformin loses its anti-tumor effects in severe combined immunodeficiency mice, suggesting the critical role of lymphocytes for these anti-tumor effects^35,36^. Metformin has also been shown to mediate the repolarization of macrophages from the M2 to M1 phenotype in the tumor microenvironment, which may lead to the inhibition of tumor growth in murine models^37–39^. More recently, metformin has been shown to downregulate programmed cell death receptor ligand-1 (PD-L1) in tumor cells^40–42^ as well as to suppress the transcription of PD-1 gene in cytotoxic T cells (CTL)^43^. These experimental results suggest that the anti-tumor property of metformin is closely related to the host immune system.

**Figure 1:**
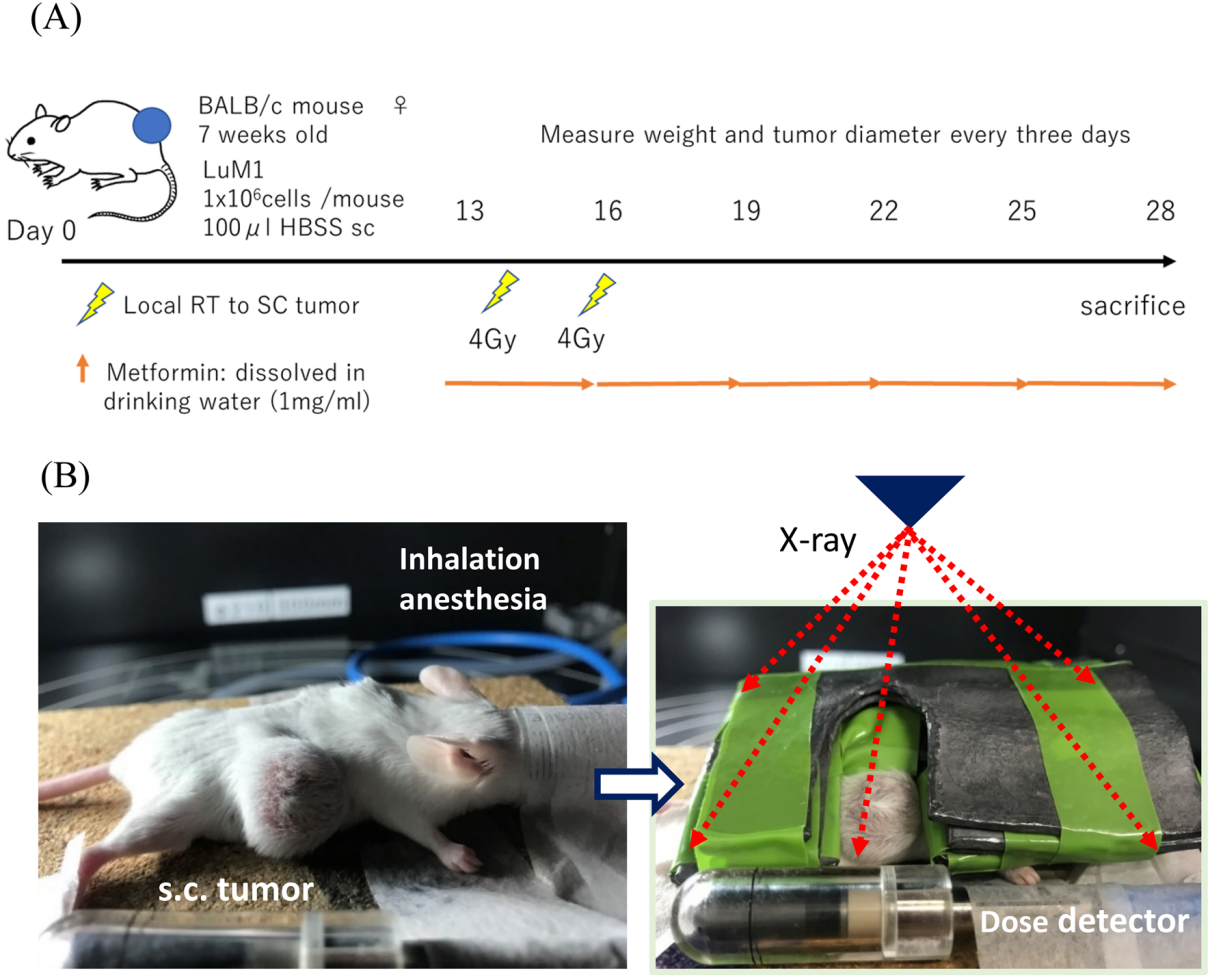
Treatment with radiation therapy (RT) and/or metformin. LuM-1 cells (1 × 10^6^ per mouse) were subcutaneously injected in the right flank of 6–7 week-old female BALB/c mice. Local RT was delivered using MX 160 Labo (mediXtec, Chiba, Japan), as described in Materials and Methods. Metformin was dissolved in the drinking water (1 mg/mL) and administrated continuously from day13.

In this study, a murine model of spontaneous lung metastases from colorectal cancer was used and the effects of combined local RT and systemic metformin on non-irradiated lung metastases examined, as well as on the irradiated subcutaneous tumor, focusing on effects on the host immune system (Fig. 1).

## Results

### Metformin augments the suppressive effects of RT on the growth of sc tumors

**Figure 2:**
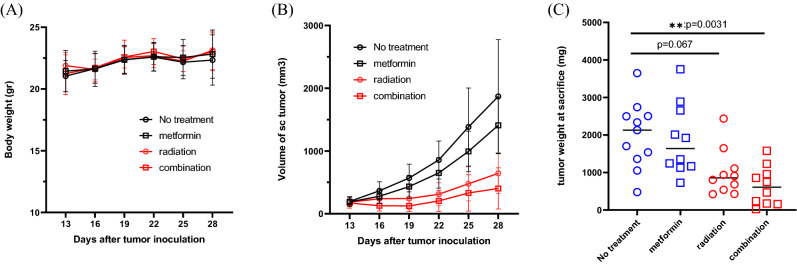
Mouse body weight (**A**) and tumor volume (**B**) were measured once every 3 days after the initiation of treatment. Mice were sacrificed on day 28 and the weight of subcutaneous tumors measured (**C**). The combined data of the results of 2 experiments (n = 5 in each) are shown: **p < 0.01.

Subcutaneous tumors were observed in all mice about 10 days after sc injection and treatment started from day 13. Weight did not show significant differences in all groups (Fig. 2A). As shown in Fig. 2B,C, local RT (4 Gy × 2) suppressed the growth of sc tumors to a size approximately half that observed in the no treatment group although not statistically significantly (*p* = 0.067). Metformin alone did not significantly suppress tumor growth. However, when combined with RT, tumor weight at day 28 was significantly reduced compared with no treatment group (*p* < 0.01, n = 10).

### Combined RT and metformin treatment suppressed the growth of non-irradiated lung metastases

**Figure 3:**
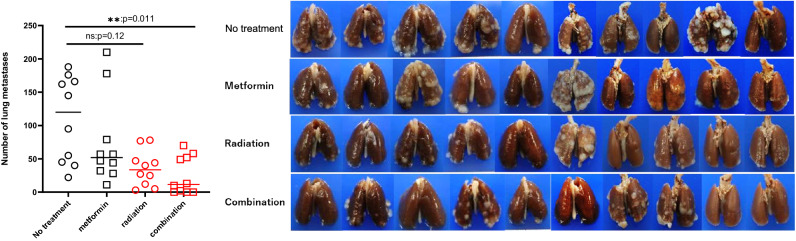
After sacrifice on day 28, the number of macroscopic metastatic nodules in the lungs evaluated. Data for the 2 experiments (n = 5 in each) are shown: **p < 0.01.

The number of macroscopic metastases in non-irradiated lungs was counted after sacrifice on day 28. As shown in Fig. 3, multiple metastases developed in the lungs of mice in the no treatment group (median (M) = 120, 22–188) which was not significantly changed by treatment with metformin alone. The number of metastases tended to be reduced in the RT treated group, although not statistically significantly so (M = 34, 3–78, *p* = 0.12). In comparison, when metformin was combined with RT treatment, the number of lung metastases was significantly decreased (M = 13, 0–70) with no metastases detected in 3/10 mice (Fig. 3). We also examined the metastatic lesions including the microscopic tumors inside the lung using microscopic observation in representative coronal tissue sections, and found that lung metastases were also suppressed significantly by combination treatment (Supplementary Fig. 1).

### Combined RT and metformin treatment increased the frequencies of T and natural killer (NK) cells in the spleens of LuM-1 bearing mice

**Figure 4:**
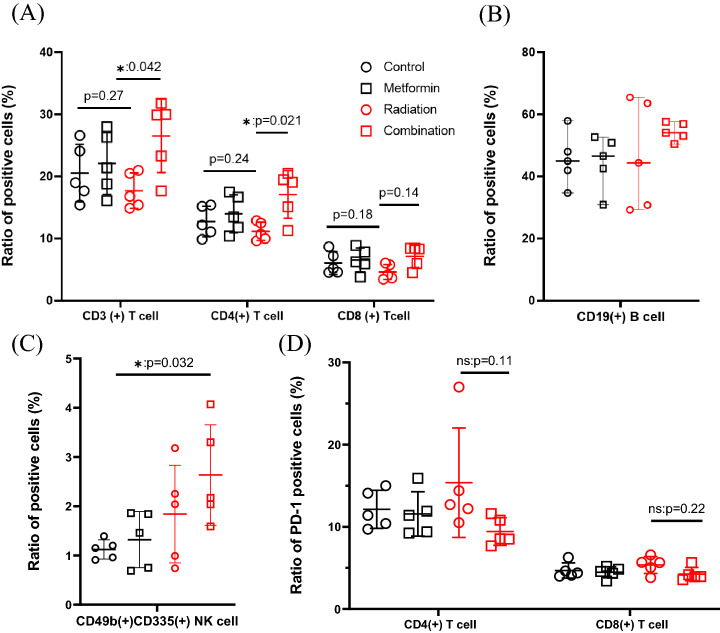
Splenocytes were harvested at day 28 and immunostained with mAbs to CD3, CD4, CD8, CD19and CD49b, CD335 and the frequencies of T (**A**), B (**B**) , activated NK (**C**) cells and PD-1(+) cells in CD4(+) or CD8(+) T cells (**D**) are determined in lymphocyte gate determined with flow cytometry. Data are shown as mean ± standard deviation of 5 mice in one of the 2 different experiments. *p < 0.05.

**Figure 5:**
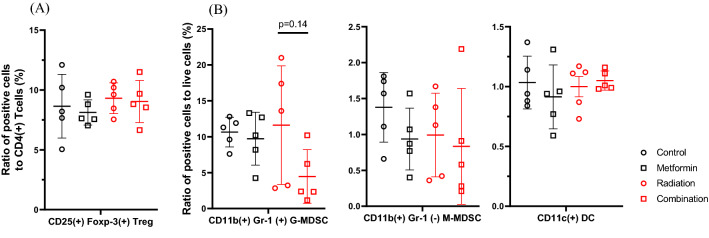
Splenocytes were harvested on day 28 and the frequencies of T-reg to CD4( +) T cell population (**A**) and CD11b(+)Gr-1(+) granulocytic-MDSC, CD11b( +)Gr-1(−) monocytic-MDSC as well as CD11c(+) DC in live cell gates (**B**) were determined with flow cytometry. Data are shown as mean ± standard deviation of 5 mice in one of the different 2 experiments.

As shown in Fig. 4A, the ratios of CD3(+) or CD4(+) T cells at day 28 tended to be reduced in mice treated with RT alone, which were recovered in mice treated with both RT and metformin. The difference between RT treated mice and the combined therapy group was statistically significant (CD3:17.8 ± 2.9% vs 26.5 ± 5.9%, *p* < 0.05; CD4:14.7% ± 1.5% vs 17.1 ± 3.8%, *p* < 0.05). The ratios of CD8(+) T cells also showed the same trend (4.6% ± 1.2% vs 7.1 ± 1.8%, p = 0.14). The rate of CD19(+) B was not different (Fig. 4B). However, the rate of activated NK cells was significantly increased in the combined therapy group (1.1% ± 0.34% vs 2.6% ± 1.0%, *p* < 0.05) (Fig. 4C). The ratios of regulatory T cells (T_reg_) and granulocytic- or monocytic-myeloid derived suppressor cells in splenocytes did not show significant differences among the 4 groups (Fig. 5).

### Combined radiation and metformin treatment restored T cell exhaustion in spleens of LuM-1 bearing mice

**Figure 6:**
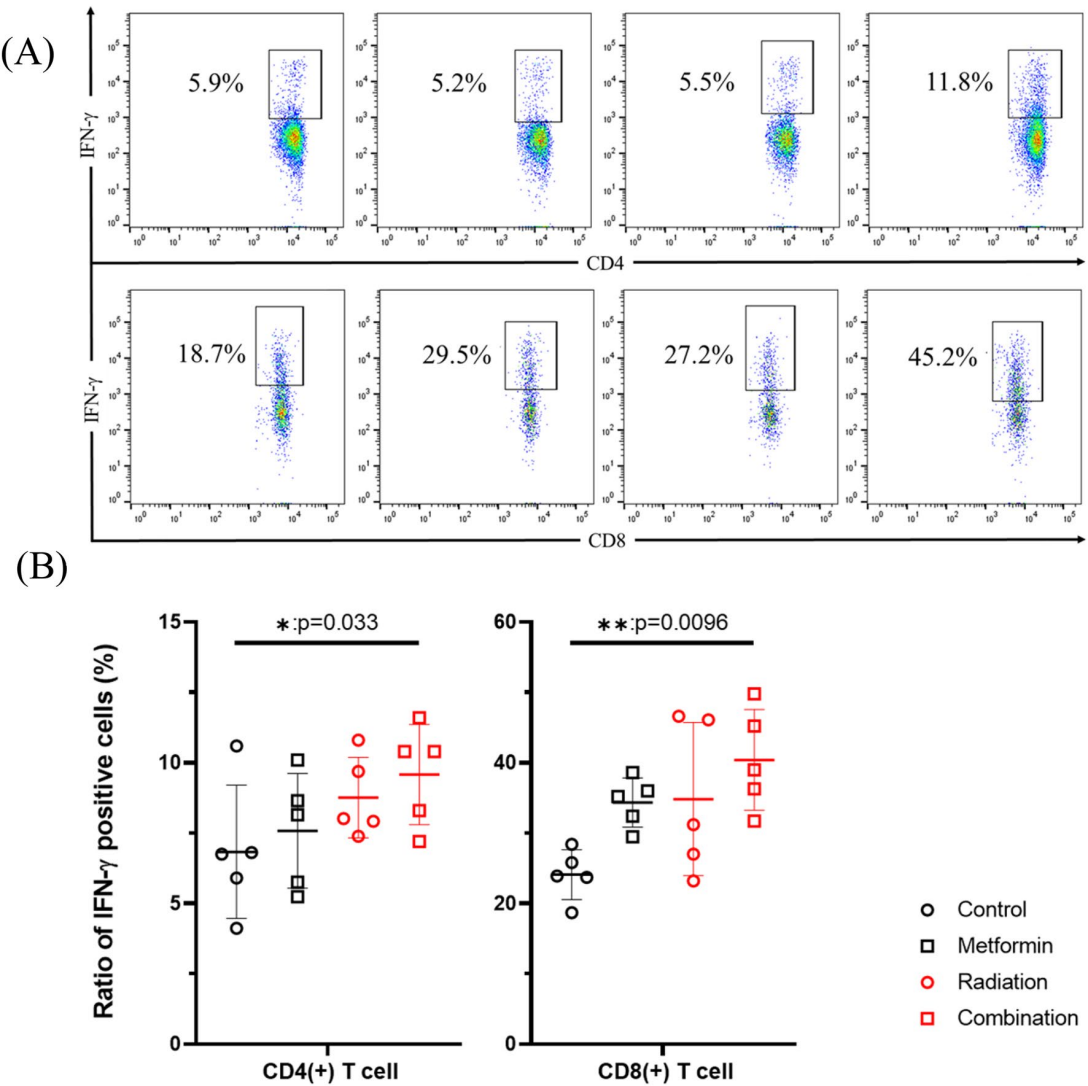
Splenocytes harvested on day 28 were cultured in the presence of 50 ng/ml PMA and 1 μg/ml ionomycin with 5.0 μl/mL brefeldin A for 4 h and cells positive for intracellular IFN-γ in these cell populations were evaluated with flow cytometry (**A**): Representative profiles of intracellular staining of IFN-γ (**B**) Data are shown as mean ± standard deviation of 5 mice in one of the 2 experiments. *p < 0.05, **p < 0.01.

We next examined T cell exhaustion in splenocytes. Although the expression of a representative exhaustive marker, PD-1 did not change significantly in CD4(+) and CD8(+) T cells, the ratios of PD-1 (+) cells tended to be lower in the combined treatment group compared with RT alone treated group (CD4;15.4 ± 6.7% vs 9.4% ± 1.7%, *p* = 0.11 CD8; 5.4 ± 1.0% vs 4.2% ± 0.80%, *p* = 0.22) (Fig. 4D). After stimulation with PMA and Ca^2+^ ionophore, the ratios of IFN-γ (+) cells were significantly increased in mice treated with both RT and metformin compared with those in the control group (CD4; 6.8 ± 2.4% vs 10.4 ± 1.3%, *p* < 0.05; CD8; 24.1 ± 3.1% vs 40.4 ± 3.1%, *p* < 0.01) (Fig. 6).

### The number of lung metastases correlates with the ratios of IFN-γ producing T and NK cells

**Figure 7:**
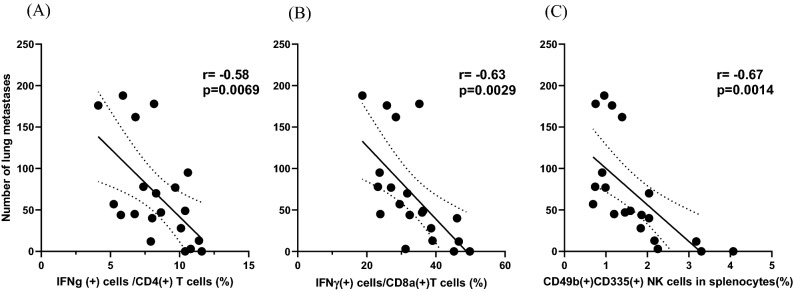
The correlation between the number of lung metastases and the ratios of IFN-γ (+) cells in CD4(+) (**A**) or CD8(+) T (**B**) cells as well as CD49b(+)CD335(+) NK (**C**) cells were evaluated with Pearson’s correlation coefficients.

Correlations between the character of splenocytes and lung metastases were examined in mice in the 4 groups. As shown in Fig. 7, the number of lung metastases was markedly reduced in mice with high ratios of IFN-γ of producing CD4(+) or CD8(+) T cells with Pearson correlation coefficients of − 0.58 and 0.63, respectively (*p* = 0.0069, 0.0029). Lung metastases showed the same inverse correlation with the frequency of CD49(+) CD335(+) NK cells (r = − 0.670, *p* = 0.0014).

### Combination therapy increased the density of NK cells while decreased the density of MDSC in lung metastases was decreased

**Figure 8:**
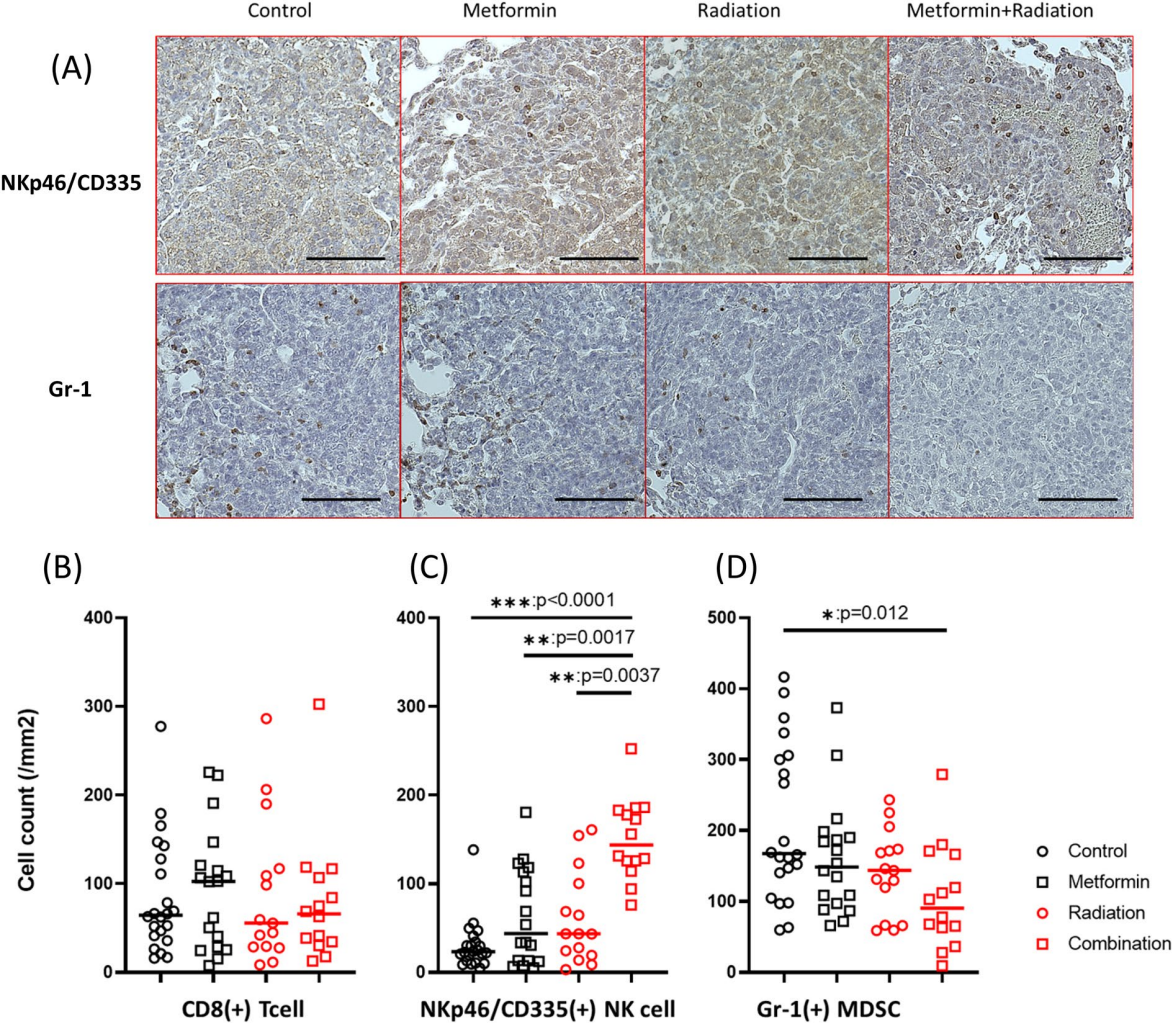
Immune cells in metastatic lesions in lung. Tissue specimens from the lungs excised at day 28 were immuno-stained with antibodies to CD8a, NKp46/CD335, or Gr-1(Ly-6G/Ly-6c) as described in Materials and Methods. (**A**) Representative immunohistochemistry of NKp46/CD335 (upper) and Gr-1 (lower) in metastatic lesions from the lung of the 4 groups. Black bars are 100 µm long. Densities of CD8(+) T cells (**B**), CD335(+) NK cells (**C**) and Gr-1(+) MDSC (**D**) infiltrating metastatic lesions in the lung. In each tissue section, the number of positively stained cells were determined in randomly selected 1–5 different metastatic lesions with more than 40,000µm^2^ area using ImageJ software. (total: 22 lesions in control, 19 in metformin, 15 in RT and 14 in combination groups). *p < 0.05, **p < 0.01, ***p < 0.001.

The character of infiltrating immune cells in metastatic lesions larger than 40,000 µm^2^ in lung tissue was evaluated with immunohistochemistry. The density of CD8a (+) cells was not changed among the 4 groups (Fig. 8B). However, as shown in Fig. 8A,C, the numbers of NKp46/CD335(+) NK cells in metastatic tumors were markedly increased only in mice treated with RT+ metformin compared with the no treatment group (*p* < 0.0001). In contrast, the density of Gr-1 (+) cells was significantly decreased in combined therapy treated group (*p* = 0.012) (Fig. 8A,D).

## Discussion

Metformin has been shown to reduce the development of various malignancies and cancer-related mortality in patients with type 2 diabetes mellitus^6–10^. In patients with colorectal cancer, metformin has been shown to improve patient outcomes although the underlying mechanisms of how metformin exerts its anti-tumor effects are not fully understood^11,44–47^. Based on these observations, many preclinical studies were conducted to determine the clinical usefulness of combined RT and metformin which have had promising results^12,16,17^. However, the results of clinical studies are still inconsistent^21,22,25^.

In previous in vivo studies^12,17^, the anti-tumor effects of metformin on irradiated tumors were evaluated in immune deficient mice. However, recent studies have suggested that the anti-tumor effects of metformin are largely dependent on host immunity^35–39,43^. In this study, therefore, we used a syngeneic murine model of spontaneous lung metastases after sc implantation of colorectal cancer tumor cells and reevaluated the synergistic effects of combined RT and metformin.

LuM-1 is a highly aggressive and non-immunogenic subclone of colon 26^48^, and metformin alone did not show significant anti-tumor effects on sc tumors or lung metastases. However, synergistic effects of RT and metformin were observed. Selective RT (4 Gy × 2) delayed the growth of sc tumors with marginal statistical significance. However, when combined with oral metformin, the tumor suppressive effects of RT were markedly enhanced. More interestingly, combined therapy suppressed the growth of metastatic tumors in non-irradiated lungs. No metastases were detected in 3/10 mice. Metastatic lesions examined with tissue sections also show the same trends. The measurement of metastases with these methodologies may not be enough to assess the accurate total metastatic burden in lung. However, microscopic metastases from the primary LuM-1 tumor were already present in the lungs at the time of starting treatment^49^, these data are highly suggestive that metformin not only enhances the cytotoxic effects on tumor cells directly exposed to RT but also causes tumor regression outside the irradiated field as a so-called “abscopal effect”^30,50^.

Although DNA double-strand breaks in tumor cells are considered to be the primary mechanism of the anti-tumor effects of RT, recent studies suggest that a reduction in tumor size is also strongly dependent on immunogenic death of tumor cells mediated by T cell-mediated host immune responses after RT^51,52^. Indeed, local RT has been shown to increase the generation of tumor antigen-specific effector T cells in murine tumor models^52,53^. In data from the present study, the rates of CD3(+), CD4(+) and CD8(+) T cells tended to be decreased in the spleens of mice treated with RT alone presumably due to direct toxic effects of RT^54^. However, the ratios were significantly increased by the additional treatment with metformin. The frequencies of IFN-γ positive cells in CD4(+) and CD8(+) T cells were significantly elevated, and PD-1(+) cells in both T cell populations tended to be reduced in mice receiving combined therapy. The results observed in irradiated mice are consistent with those from previous studies showing that the anti-tumor effects of metformin result mostly from T cell mediated host immunity^35,36,43^. The IFN-γ producing activities of T cells is critical to suppress the growth of metastases since they inversely correlate with the degree of lung metastases in the mice in all 4 groups in the present study. Taken together, it is suggested that RT alone may reduce systemic T-cell mediated immunity associated with a non-immunogenic tumor such as LuM-1, while metformin restore T cell exhaustion which can efficiently induce abscopal effects.

Another interesting finding is that the frequency of activated NK cells defined by the CD49(+) CD335(+) phenotype showed a strong inverse correlation with the number of lung metastases and their ratios were significantly increased in mice treated with combined RT and metformin. Immunohistochemical analysis revealed that the number of CD335(+) activated NK cells, but not CD8(+) T cells, infiltrating metastatic lesions was increased in those mice. Consistent with this result, a recent study has demonstrated that metformin increases the frequency of CD335(+) NK cells in the spleen which suppresses pulmonary metastases from B16F10 melanoma^55^.

However, infiltration of Gr-1(+) MDSC in lung metastases was reduced after combined treatment. Numerous studies have suggested that accumulation of MDSC provide a tumor permissive microenvironment through stimulation of angiogenesis as well as potent suppression of T cell functions^56,57^. MDSC have also been reported to inhibit NK cell functions^58–60^, although the mechanistic interactions with NK cells are less clear than with T cells^61^. Considering the results of these studies, it is suggested that the abscopal effects induced by metformin and RT might be partially attributed to augmented NK cell activity caused by a reduced number of MDSC in the lung.

There is growing evidence that RT can result in in situ tumor vaccination by exposing tumor specific neoantigens to the host’s innate immune system which then leads to immunogenic cell death of non-immunogenic tumor cells^51–53,62^. After successful results were found with immune check point inhibitors (ICI) in the treatment of various malignancies, radioimmunotherapy using the combination of RT and ICI has attracted much attention as an effective novel therapy for advanced malignancies. However, studies have shown conflicting results of the clinical usefulness of combined RT and ICI presumably due to differences in cancer immunogenicity as well as host immunity^31–34^. The present study shows that metformin efficiently enhances the abscopal effects of RT in non-immunogenic colorectal cancer tumors though the activation of T cell- and NK cell-mediated immunity. The results may not directly apply to humans. However, since metformin activates anti-tumor immunity by different mechanisms from anti-PD-1/PD-L1 or anti-CTLA-4 mAbs, the combination of immune check point inhibitors and metformin may provoke remarkable radiosensitizing effects leading to improved outcomes in patients with LARC.

## Materials and methods

### Reagents and antibodies

Metformin hydrochlorides was purchased from FUJIFILM-Wako Chemical (Osaka, Japan). Anti-mouse mAbs for flowcytometric analysis were used as described. FITC-conjugated anti-CD4(GK1.5), anti-CD8a(53-6.7), anti-CD49b(DX5), anti-Ly6c(HK1.4), and PE-conjugated anti-CD8a(53-6.7), anti-CD11c(N418), anti-CD19(6D5), anti-CX3CR1(SA0111F1), anti-IFN-γ (XMG1.2), and APC-conjugated anti-CD3(17A2), anti-CD11b(M1/70), anti-CD335(29A1.4), APC/Fire 750-conjugated anti-CD25(PC61), anti-CD45(30-F11), and BV421-conjugated anti-CD4(GK1.5), anti-FoxP3(MF-14), anti-Gr-1(RB6-8C5), anti-CD279(PD-1)(29F.1A12), and BV785-conjugated anti-CD45(30-F11), and pacific blue-conjugated CD45(30-F11) were purchased from BioLegend Inc. (San Diego, CA USA). FcR blocking reagent was purchased from Miltenyi Biotec B.V.& Co. KG (Bergisch Gladbach, Germany). FVS-780 and FVS-700 and Zombie UV were purchased from BD Biosciences (Franklin Lakes, NJ USA) and BioLegend Inc. (San Diego, CA USA), respectively. PMA and ionomycin, and Brefeldin A solution(1000X) were purchased from FUJIFILM (Osaka, Japan) and BioLegend Inc., (San Diego, CA USA), respectively. For Immunohistochemistry, mAbs to CD8a (4SM15, Rat IgG2a) and Ly-6G/Ly-6c (RB6-8C5, Rat IgG2b) were purchased from Invitrogen (Santa Clara, CA), and polyclonal Ab to NKp46/CD335 (EPR23097-35, Rabbit IgG) were from Abcam (Cambridge, MA).

### Cell lines

LuM-1, a highly metastatic sub-clone of murine colon cancer, colon26^63^ was kindly obtained from Dr. Oguri, Aichi Cancer Center, Japan, and maintained in RPMI supplemented with 10% FCS, 100 U/mL penicillin and 100 μg/mL streptomycin (Sigma-Aldrich, St. Louis, MO, USA). After achieving > 80% confluence, cells were removed by treatment with TrypLE Express(Gibco), and then used for experiments. Cultured cells were tested using the Mycoplasma Detection Kit (R&D Systems, Minneapolis, MN, USA) every 3 months and used for experiments after three passages.

### Animal model

The experimental protocol is shown in Fig. 1A. Female BALB/c mice 5–6 weeks old were purchased from CLEA Japan. (Tokyo, Japan) and housed in specific pathogen-free conditions. LuM-1 cells (1 × 10^6^ per mouse) were injected subcutaneously in the right flank of 6–7 weeks-old female BALB/c mice. When the primary tumors reached a volume of 100–300 mm^3^ at day13, mice were divided into four groups, each group containing 5–6 mice to enable statistical analysis (Group A, no treatment; Group B, metformin treated; Group C, radiation treated; Group D, metformin and radiation treated). Local RT was delivered using MX 160 Labo (mediXtec, Chiba, Japan), as described previously^49^. In short, anesthetized mice were held in the decubitus position, and radiation delivered only to the subcutaneous (sc) tumor with the remainder of the mouse including the lung covered with 3 lead plates with a thickness of 5 mm (Fig. 1B). We confirmed the X-ray was completely blocked by this apparatus. As a control, mice were similarly placed in the same conditions under anesthesia. Mice were administrated metformin hydrochloride (Wako) (1 mg/mL) or as indicated, dissolved in drinking water. Tumor diameter was measured with calipers and tumor volume calculated using tumor volume (mm^3^) = [tumor length (mm) × tumor width (mm)^2^]/2 and measured once every 3 days. Mouse weight was measured every 3 days. Mice were sacrificed using deep anesthesia by isoflurane on day 28, and the weight of the sc tumor and numbers of macroscopic metastatic nodules in the lungs were blindly evaluated by 2 investigators and average of the values were adopted. All procedures were approved by the Animal Care Committee of Jichi Medical University (No 19035-01) and performed in accordance with ARRIVE guidelines and the Japanese Guidelines for Animal Research.

### Flow cytometry

For fluorescence activated cell sorting analysis, spleens were harvested and single cell suspensions prepared. After passing through 70 μm nylon mesh, red blood cells were eliminated with lysis buffer (NH4Cl, KHCO3, EDTA 4Na) and single cell suspensions prepared. Splenocytes were adjusted to 1X10^6^ cells in PBS containing 0.02% EDTA and incubated for 15 min to label dead cells. After washing with PBS, the cells were incubated with 5 μl FcR blocking reagent for 10 min and immunostained with relevant mAbs according to manufacturer’s instructions. Intracellular staining of Foxp3 (BV421, BioLegend, clone MF-14) was performed using True-Nuclear Transcription Factor Buffer Set (BioLegend, San Diego, CA). After washing with PBS, the cell suspension was applied to BD LSRFortessaTM X-20 (Becton–Dickinson, San-Jose, CA USA) and antigen expression analyzed using Flow Jo TM software (Becton–Dickinson, San-Jose, CA USA). T cells producing IFN-γ were identified by intracellular staining. Prepared splenocytes (1 × 10^6^) were cultured in RPMI-1640 + 10% FCS for 4 h at 37 °C in the presence of 50 ng/ml PMA (Wako Chemical) and 1 μg/ml ionomycin (Wako Chemical) and 5.0 μl/mL brefeldin A (BioLegend). The cells were harvested, fixed, permeabilized using the fixation and permeabilization solution (BD Bioscience) according to the manufacturer’s instructions and stained with PE-conjugated IFN-γ or isotype control Rat IgG1 and FITC-conjugated anti-CD8a, APC-conjugated anti-CD3 and BV421-conjugated anti-CD4 mAb as well as FVS780 to exclude dead cells. The ratio of IFN-γ positive cells was calculated in CD3(+)CD4(+) or CD3 (+)CD8a (+) gated areas using LSR FortessaTM X-20 (BD Bioscience).

### Immunohistochemistry of mice lung samples

Mice were sacrificed at day 28, lungs were internally fixed with 4% formalin, excised, and paraffin-embedded 4 µm sections were prepared for immunohistochemical evaluation. After endogenous peroxidase blocking by methanol and 30% hydrogen peroxide and heat-induced antigen retrieval in citrate buffer with microwaves, the specimens were incubated with 1% BSA for 30 min to block nonspecific antibody binding. Then, the slides were incubated with Abs to CD8a (at a dilution of 1:100), Ly-6G/Ly-6c (1:50), or NKp46/CD335 (1:500) in humid chambers overnight at 4 °C. After washings with PBS, sections were incubated with anti-rat or anti-rabbit secondary antibody conjugated with peroxidase for 30 min at room temperature. After washing, the enzyme substrate 3, 30-diaminobenzidine (Dako REAL EnVision Detection System, DAKO) was used for visualization and counterstained with Meyer’s hematoxylin. In each tissue section, 1–5 different metastatic lesions larger than 40,000 µm^2^ were randomly selected and positively stained cells were counted in those areas under the microscope. Thereafter, the densities of each cell type (/mm^2^) were calculated using ImageJ software (NIH, Bethesda, MD).

### Statistical analysis

For data on tumor volume, lung metastases and immunohistochemistry, *p*-values were evaluated with Kruskal–Wallis analysis followed by Dunn’s multiple comparison tests. For splenocyte data, *p*-values were evaluated with one-way ANOVA followed by Tukey’s honestly significant difference test. Correlation was examined with simple linear regression analysis. All analyses were performed with Graph Pad Prism 8 Software (San Diego, CA, USA), and *p*-values < 0.05 were considered statistically significant.

## Supplementary Information

Supplementary Figure 1.

Supplementary Information.

## Data Availability

All data generated or analysed during this study were included in this published article and its supplementary information files.
