# Supplementary figures and images for: Metformin combined with local irradiation provokes abscopal effects in a murine rectal cancer model
Source: Sci Rep. 2022 May 4;12:7290. doi: 10.1038/s41598-022-11236-2 (PMC9068771; doi:10.1038/s41598-022-11236-2)

## Slide 1
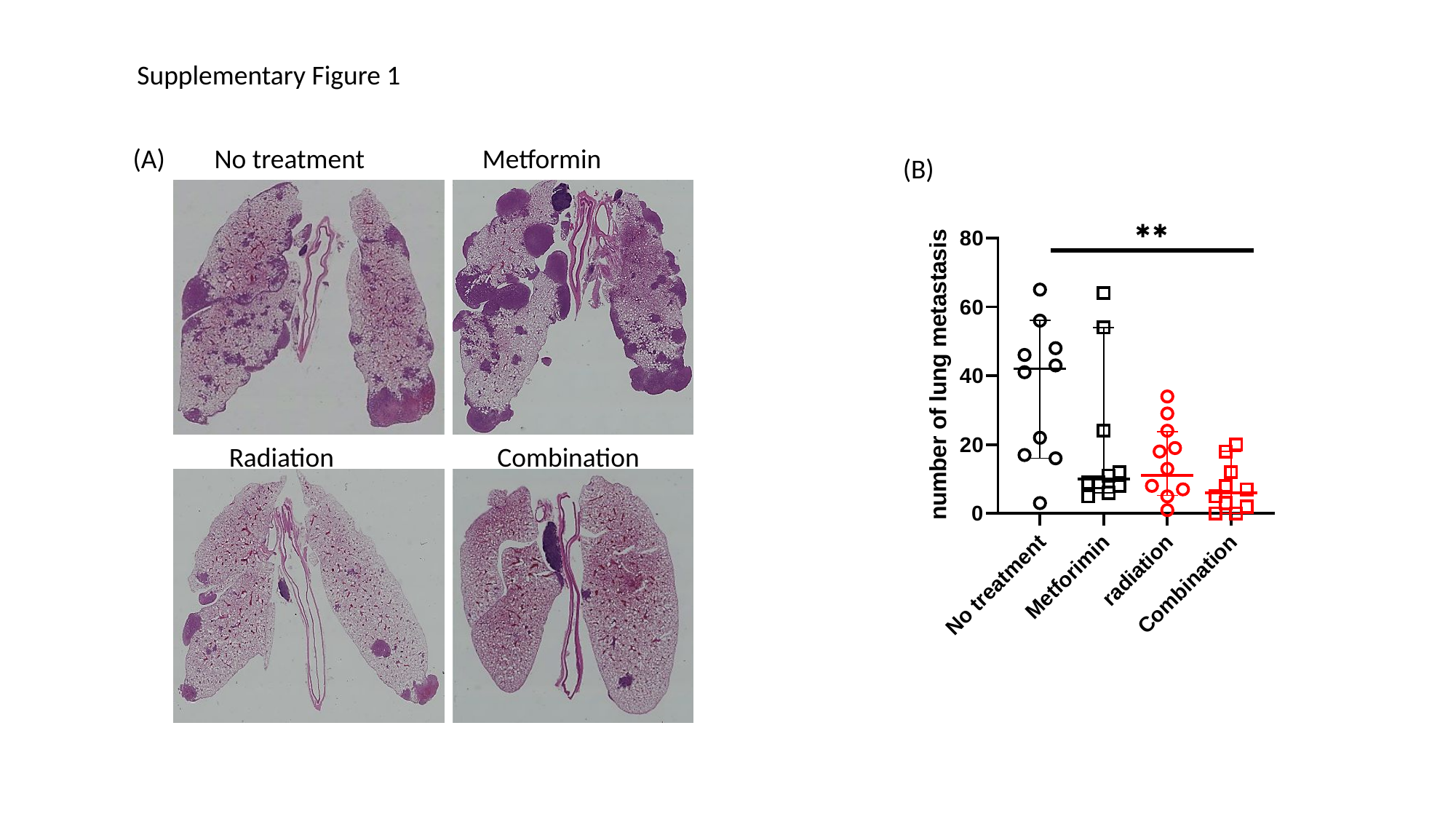

Supplementary Figure 1
(A)
No treatment
Metformin
(B)
Radiation
Combination

Supplement: Supplementary file 1 — Supplementary Figure 1. [file 41598_2022_11236_MOESM1_ESM.pptx]
