# Supplementary material for: Metformin combined with local irradiation provokes abscopal effects in a murine rectal cancer model
Source: Sci Rep. 2022 May 4;12:7290. doi: 10.1038/s41598-022-11236-2 (PMC9068771; doi:10.1038/s41598-022-11236-2)
Supplement: Supplementary file 2 — Supplementary Information. [file 41598_2022_11236_MOESM2_ESM.docx]

Supplementary Figure 1 Coronal tissue sections in the central part of the formalin-fixed lung specimens were stained with Hematoxylin-Eosin staining (A) and the total number of metastatic tumors including microscopic lesions in lung were counted under the microscope (B). Counting was blindly performed with 2 different investigators and average values were adopted. **:*p* < 0.01.
